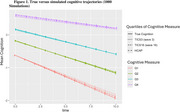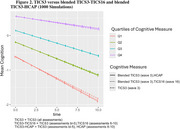# The consequences of changes to neuropsychological test batteries on measuring cognitive decline in long‐running studies of cognitive aging and dementia

**DOI:** 10.1002/alz70860_103657

**Published:** 2025-12-23

**Authors:** Ryan M Andrews, Brandon E Gavett, Natalia Gomes Goncalves, Emma Nichols, Kendra D Sims, Douglas Tommet, Yingyan Wu, Dan M. Mungas, Kelvin P Zhang

**Affiliations:** ^1^ Columbia University, New York, NY, USA; ^2^ University of California Davis, Sacramento, CA, USA; ^3^ University of São Paulo Medical School, São Paulo, SP, Brazil; ^4^ University of Southern California, Los Angeles, CA, USA; ^5^ Boston University, Boston, MA, USA; ^6^ Brown University School of Medicine, Providence, RI, USA; ^7^ UCLA Fielding School of Public Health, University of California, Los Angeles, CA, USA; ^8^ University of California, Davis, Sacramento, CA, USA; ^9^ University of Michigan, Ann Arbor, MI, USA

## Abstract

**Background:**

Several large, representative studies of older adults, like the US Health and Retirement Study (HRS), have collected longitudinal cognitive data on their participants; however, the cognitive batteries used by the HRS have changed over time, including the introduction of the Harmonized Cognitive Assessment Protocol (HCAP) battery for a subset of HRS participants. When cognitive batteries change over time, changes in measurement properties might result in misleading findings about cognitive trajectories. The aim of this study was to use simulation methods to assess the magnitude of bias in estimating cognitive decline that is induced by test batteries that changed over time.

**Method:**

We simulated true cognition using non‐linear models of cognitive change derived from four harmonized longitudinal cognitive aging studies (UC Davis Alzheimer’ Disease Research Center longitudinal cohort, Kaiser Healthy Aging and Diverse Life Experiences study, Kaiser Study of Healthy Aging in African Americans, Kaiser Life After 90 Study). Empirical HRS‐HCAP item parameters were used as true item parameters, and item response theory methods were used to simulate measured test results for different batteries of cognitive tests in HRS and HRS‐HCAP. We estimated “blended” cognitive trajectories, artificially introducing mid‐course changes of the simulated test used to measure cognition. To illustrate the impact of these changes, we then used linear mixed‐effects models to estimate 11‐year cognitive trajectories, overall and by quartile of true decline.

**Result:**

Using instruments with the highest measurement precision led to estimated cognitive trajectories that best matched the truth. At the same time, estimated trajectories using less informative test versions were very closely related (Figure 1), such that blended trajectories did not deviate from the truth substantially (Figure 2).

**Conclusion:**

Our results support the use of high‐quality instruments, like the HCAP battery, for optimally studying cognitive trajectories. However, differences between estimated HCAP and HRS‐TICS trajectories were small, suggesting that, in practice, changes in test batteries over time may not meaningfully affect estimates of cognitive decline.